# Natural Killer Cells and Type 1 Innate Lymphoid Cells Are New Actors in Non-alcoholic Fatty Liver Disease

**DOI:** 10.3389/fimmu.2019.01192

**Published:** 2019-05-28

**Authors:** Carmelo Luci, Elodie Vieira, Thibaut Perchet, Philippe Gual, Rachel Golub

**Affiliations:** ^1^Université Côte d'Azur, INSERM, Centre Méditerranéen de Médecine Moléculaire, Nice, France; ^2^Unité Lymphopoïèse, Institut Pasteur, INSERM U1223, Université Paris Diderot, Paris, France

**Keywords:** NAFLD, NASH, NK cells, ILC1, liver, inflammation

## Abstract

Obesity and associated liver diseases (Non Alcoholic Fatty Liver Disease, NAFLD) are a major public health problem with increasing incidence in Western countries (25% of the affected population). These complications develop from a fatty liver (steatosis) to an inflammatory state (steatohepatitis) evolving toward fibrosis and hepatocellular carcinoma. Lipid accumulation in the liver contributes to hepatocyte cell death and promotes liver injury. Local immune cells are activated either by Danger Associated Molecular Patterns (DAMPS) released by dead hepatocytes or by bacterial products (PAMPS) reaching the liver due to increased intestinal permeability. The resulting low-grade inflammatory state promotes the progression of liver complications toward more severe grades. Innate lymphoid cells (ILC) are an heterogeneous family of five subsets including circulating Natural Killer (NK) cells, ILC1, ILC2, ILC3, and lymphocytes tissue-inducer cells (LTi). NK cells and tissue-resident ILCs, mainly located at epithelial surfaces, are prompt to rapidly react to environmental changes to mount appropriate immune responses. Recent works have demonstrated the interplay between ILCs subsets and the environment within metabolic active organs such as liver, adipose tissue and gut during diet-induced obesity leading or not to hepatic abnormalities. Here, we provide an overview of the newly roles of NK cells and ILC1 in metabolism focusing on their contribution to the development of NAFLD. We also discuss recent studies that demonstrate the ability of these two subsets to influence tissue-specific metabolism and how their function and homeostasis are affected during metabolic disorders.

## Introduction

**Figure 1:**
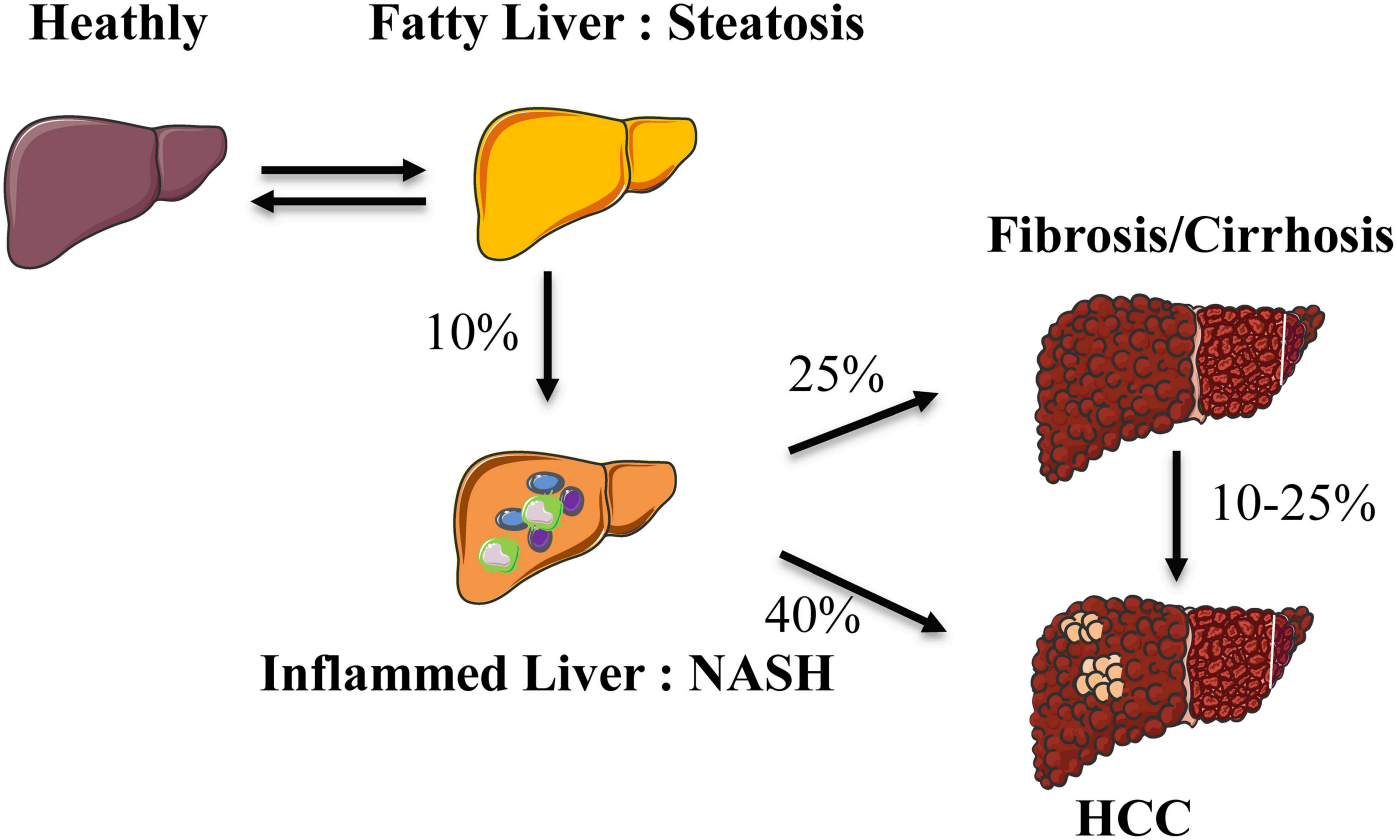
Spectrum of hepatic abnormalities in NAFLD. During obesity, liver complications develop from a fatty liver (steatosis) to an inflammatory state (steatohepatitis) evolving toward fibrosis and hepatocellular carcinoma. NASH, non-alcoholic steatohepatitis; HCC, hepatocellular carcinoma.

Non-alcoholic Fatty Liver Disease (NAFLD) has recently become a pathology of interest as its increased prevalence is associated with obesity and metabolic syndrome. The spectrum of the hepatic disease ranges from steatosis (fatty liver) to steatohepatitis (Non-alcoholic Steato-Hepatitis, NASH) and then the activation of fibrogenic pathways. NASH represents the progressive form of NAFLD and predisposes to more severe end-stage liver disease such as cirrhosis and/or hepatocellular carcinoma (Figure 1) (1).

The chronic low-grade inflammation associated with obesity plays an important role in development and the progression of NAFLD. These hepatic diseases now represent the most frequent hepatic lesion in western countries (2). Hepatic steatosis is the initial step of the disease characterized by an influx of lipids coming from inflamed adipose tissue (AT), increased hepatic *de novo* lipogenesis and decreased lipid catabolism. Lipid accumulation in hepatocytes (free fatty acids, cholesterol…) leads to lipotoxicity with sustained oxidative and endoplasmic reticulum stresses making them more susceptible to cell death. Liver injury and the activation/recruitment of inflammatory immune cells promote disease progression to NASH. The molecular mechanisms responsible for the progression from a “non-pathogenic” steatotic state to NASH are still unclear. Despite lifestyle changes, the treatment of NASH is still limited because of the lack of effective pharmacological treatment as well as lack of effective and practical diagnostic tools. Recent findings suggest that pathogenesis of NASH involves cross talks between liver, gut and adipose tissue into which the physiology of resident/recruited immune cells is affected.

The liver is exposed to a variety of harmless (dietary products) and harmful (pathogens, toxins) particles coming from the gastrointestinal tract. The liver immune system maintains an equilibrium between immune surveillance against pathogens and tolerance to innocuous antigens and commensal bacteria. This equilibrium is altered during obesity leading to low grade inflammation that promotes the progression of liver diseases. Inflammation has thus become a prominent player in the pathological mechanism of NALFD (3). Obesity is associated with alterations of the gastrointestinal flora (dysbiosis), increased gut permeability and afflux of free fatty acids from AT into the liver. Translocation of bacterial products via the portal vein triggers low-grade inflammation by acting on innate immune cells and play an important role in the progression of fatty liver to an inflammatory state. Hepatic accumulation of lipids is the hallmark of NAFLD that may influence its progression. Specific lipid species can cause toxicity promoting hepatocytes death which in turn release DAMPs that may activate local immune cells such as macrophages, NK-T cells, and adaptive T cells (4, 5).

Innate lymphoid cells (ILC) are the most recently discovered family of immune cells that are mainly localized at epithelial surfaces where they maintain tissue homeostasis. Their strategical locations allow them to rapidly respond to pathogen invasion by mounting appropriate immune responses. ILC are non-T, non-B lymphocytes that lack antigen receptors encoded by rearranged genes (6, 7). Similarities between ILC and T cell subsets led to propose ILC as the innate counterparts of T cell subsets. Even if many functions of ILC are largely redundant with those provided by T cells (8), both populations are important coordinators of the immune response by integrating environmental signals and by interacting with numerous types of cells (9). A fresh nomenclature revisits the previous classification of the ILC family based on cell surface markers, transcription factor requirement and ability to produce type 1, type 2, and Th17 cell-associated cytokines and now distinguishes it into five subsets: NK, LTi, ILC1, ILC2, and ILC3 (10, 11). ILC have been involved in immune responses against bacteria, virus, parasites, allergens, and tumor (10–12). The recent progress in the ILC field led to the identification of new ILC subsets in tissues harboring metabolic function such as in liver and adipose tissue (13). The interest in the crosstalk between host metabolic environment and ILC in specific tissues is growing. How the metabolic changes and dysbiosis associated with NAFLD impact on ILC differentiation and functions is still an open question. In this review, we evaluate the potential role of NK cells and ILC1 in the pathophysiology of NAFLD.

## Development and Specific Properties of Ilc1 and NK Cells

The five ILC subsets derive from the common lymphoid progenitor that progresses toward the early innate lymphoid progenitor in parallel to the upregulation of CXCR6 and the α4β7 integrin expression (14, 15). The further stage called common helper ILC precursor contains precursors restricted to ILC1, ILC2, and ILC3 lineages (16). The position of NK and LTi lineages along the ILC developmental scheme raised some controversies with data in favor of a lineage commitment preceding the acquisition of functions. Notably, the expression of PLZF transcription factor is a key step in the divergence of these fates (17, 18).

Conventional NK cells (NK cells) and helper-like ILC1 could be discriminated by the identity of their T-box transcription factors. The T-box protein in T cells, Tbet, encoded by the *Tbx21* gene is involved in IFN-γ production. Eomesodermin (Eomes) transcription factor shares homology with Tbet. Mature NK cells are Tbet^+^ Eomes^+^ while ILC1 are Tbet^+^ Eomes^−^. In the liver, Tbet upregulation is induced with ILC1 differentiation (19, 20). Studies using Eomes reporter mice proved that despite their immature phenotype, Tbet^+^ hepatic ILC1 are not precursors of Eomes^+^ NK cells (20). ILC1 and NK lineages have been shown to diverge early in ontogeny (17).

A great diversity was described for NK cells and ILC1 that differentially expressed a large number of markers depending on the organ and the activation state (21). Resident ILC1 subsets have also been defined in multiple organs and are enriched in liver, gut, uterus, skin and salivary glands (22). An absence of commonly conserved unique markers among ILC1 prevents from a common definition and suggests tissue specific functions. On top of differential Eomes expression, distinction between NK cells and ILC1 also concerns their cytotoxic properties and resident/tropic molecule expression. Relationships between NK and ILC1 lineages are still fuzzy since distinctive markers are either not supported in few tissues or after inflammatory conditions. Indeed, while Eomes expression is restricted to NK cells in most organs, it could be expressed by salivary gland ILC1 (23). CD127 is expressed by most ILC despite a disparate expression among hepatic ILC1 and an absence from those of the salivary glands. Following activation, NK cells could start to express CD49a and CXCR6, as ILC1. Hence, transitional subsets expressing different levels of CD49a and Eomes in case of inflammation, chronic disease or in tumor, strengthen their plastic properties (24, 25). Furthermore, the inhibitory receptor CD200r1 is expressed on the cell surface of peripheral ILC1 (into liver, adipose tissue, small intestine) but not on NK cells (26). It has been suggested that the combined expression of CD200r1, Eomes, with CD49b could differentiate ILC1 from NK cells during homeostasis and viral-induced inflammation (26). To note, other ILC population such as ILC2 and ILC3 in the adipose tissue and small intestine also expressed CD200r1 suggesting that CD200r1 may help to discriminate NK cells from all other ILCs. The mechanisms regulating T-box transcription factor expression in different organs are mostly unknown. The Notch signaling pathway can regulate the Tbet/Eomes ratio and the pro-inflammatory cytokine expression in hepatic NK and ILC1 subsets (27). Moreover, the ectopic expression of Eomes in ILC1 induced their conversion toward mature NK cells, suggesting a role for Eomes in plasticity (28). On the other hand, NK cells and ILC1 share NKp46, NK1.1, NKG2D, and CD122 surface makers that were routinely used in most studies without specific evaluation of the NK/ILC1 input. Both intestinal and hepatic ILC1, are Eomes^−^Tbet^+^NK1.1^+^ cells requiring Tbet to develop (16, 20). Contrary to other tissues, liver ILC1 depend on the transcription factor hobit for tissue retention as tissue resident T lymphocytes (29). On top of a common CXCR6^+^CD69^+^CD49a^+^ phenotype, hepatic ILC1 also express molecules important for their tissue location and maintenance like CXCR3, CD103, CD39 or CX3CR1 (29, 30). The CD49a^+^ ILC1 population is also currently called tissue-resident NK cells (trNK cells) and is present in different tissues (skin, intestine, salivary glands, kidney, uterus, adipose tissue) with striking phenotypic similarities and some functional differences between organs that might reflect the impact of microenvironment (31) or the existence of divers subsets. In particular, liver resident NK cells/ILC1 expressed higher levels of TRAIL and FasL with stronger cytotoxic properties than their small intestine counterpart (32). Contrary to other tissues, hepatic and intestinal ILC1 are not dependent on IL7 as their total number in both organs are identical between Il7rα^−/−^ and WT mice (16, 20). In these organs, ILC1 development depend on IL-15 but not IL-7 as already demonstrated by pionner studies for NK cells (33, 34). ILC1 express high levels of IL12rβ1 and respond to IL-12 and IL-15 stimulation by the secretion of IFN-γ and/or TNF-α. IL-18 co-stimulation significantly increases IFN-γ levels (20, 35). The human CD127^+^ subset of ILC1 only produce IFN-γ contrary to its CD127^−^ counterpart producing both TNF-α and IFN-γ. Human intestinal ILC1 are mostly defined as non-cytotoxic and could impart Th1 chemoattraction and maturation partly by CXCR3 expression (36).

## NK Cells and ILC1 in Adipose Tissue Inflammation in Nafld

The AT functions encompass energy storage and endocrine activities to maintain metabolism homeostasis. The AT is composed of adipocytes, stromal cells including resident immune cells and it is now considered as a fully immunocompetent organ. In the context of obesity, AT hypoxia reflects hypertrophy and hyperplasia of adipocytes with an impaired insulin signaling, increased cell death and release of DAMPs. These stress responses allow the local accumulation and activation of pro-inflammatory immune cells (M1 macrophages, ILC1, CD4^+^ Th1, and CD8^+^ T cells). These cells sustain inflammation and lead to insulin resistance and metabolic dysregulation (37).

Adipose tissue macrophages (ATMs) represent almost 50% of the resident AT immune cells and highly contribute to low grade inflammation and insulin resistance during obesity. Indeed, ATMs are the dominant source of pro-inflammatory cytokines (TNF-α, IL-1β). The resulting AT lipolysis increases the rate of circulating free fatty acids which accumulated into the liver contributing to hepatic steatosis. The tight control of M2 to M1 transition represents a key event to regulate AT homeostasis at steady state and during diet-induced obesity (DIO) (1). Experimental evidences suggested that T-cell subsets have regulatory potential of M2/M1 balance during obesity. Indeed, AT infiltrating CD8^+^ T cells contribute to M1 macrophages phenotype whereas the decreased number of CD4^+^ regulatory T cells fosters AT inflammation in obese subjects (38, 39). Recently, it has been reported that type 1 innate lymphoid cells (T1-ILC) also contribute to the homeostasis of ATMs in lean and obese conditions. The phenotypic and functional heterogeneity of T1-ILC has been recently investigated in human and mice. All human lean adipose depots contain T1-ILC ranging from 5 to 10% among resident lymphocytes. In mice, T1-ILC represented nearly 22 to 30% of AT lymphocytes and constituted the most abundant lymphocyte population in AT (40, 41). Murine T1-ILC is composed of helper-like tissue resident ILC1 (mixed Eomes^−^ CD49a^+^ CD49b^−^ and Eomes^+^ CD49a^+^ CD49b^+^), immature (Eomes^+^ CD49a^−^ CD49b^−^) and circulating mature NK cells (Eomes^+^ CD49a^−^ CD49b^+^) and human AT is composed of two populations CD56^dim^ CD16^−^ and CD56^dim^ CD16^+^ T1-ILC1. High fat diet (HFD) challenge increases both ILC1 number and activation in AT and are the major source of IFN-γ, TNF-α. In turn, ILC1 enable ATMs-M1 polarization leading to AT inflammation and insulin resistance (40, 42, 43). Moreover, T1-ILC depletion ablation in DIO mouse model was associated with decreased macrophage infiltration, M1 polarization and insulin resistance (42–44). Boulenouar et al. reported that all the three T1-ILC (CD49a^+^ mixed ILC1, iNK cell, and mNK cell subsets) are able to preferentially kill M2 macrophages in lean conditions but have impaired cytotoxicity with obesity that may explain an increase in M2 conversion/polarization into M1 macrophages with obesity (41). Furthermore, ILC1 produce the macrophage chemoattractant CCL2 and ATMs increase their NK cell chemoattractant production such as CCL3, CCL4, and CXCL10 (43). While the upstream signals leading to obesity-associated inflammation remains to be fully defined, IL-12 was suggested as a driver of ILC1 accumulation and activation in AT (40). Therefore, the T1-ILC/-macrophage cross-talk is a key regulator of AT inflammation representing a promising therapeutic target for treating patients with metabolic syndrome and NAFLD.

## NK Cells and ILC1 in Gut Functions in Nafld

The close anatomical proximity of the gastrointestinal tract and the liver creates a functional link between these two organs. The portal venous system drains intestinal nutrients and products to the liver. During obesity, gut dysbiosis with increased intestinal permeability contribute to the development and progression of NAFLD in mouse and human (45, 46). Murine intestinal ILC1 encompassed intraepithelial ILC1 (CD49a^+^ Eomes^+^) and lamina propria-resident ILC1 (CD49a^+^ CD127^+^ Eomes^−^) which are distinct from conventional lamina propria NK cells (CD49a^−^ Eomes^+^ CD127^−^) (47). The intestinal ILC1 population is more complex that initially supposed. By using RORγt-fate map (fm) mice, Klose et al. identified a small fraction of Lin^−^ NKp46^+^ NK1.1^+^ intestinal cells (20%) originated from the ILC3 lineage, now referred to ILC1 “ex-RORγt ILC3” (16). The plasticity observed between ILC1 and ILC3 lineages was studied using mouse genetic lineage-tracking experiments and human *ex-vivo* intestinal cell cultures (48–50). Both small intestinal NK cells and NKp46^+^ ILC3 are activated after oral *Listeria monocytogenes* infection. However, only NK-derived IFN-γ contributes to limit bacterial dissemination while IL-22 produced by NKp46^+^ ILC3 appeared dispensable (51). Moreover, ILC1 have been reported as the dominant innate source of IFN-γ and TNF-α in mice infected with the *Toxoplasma gondii* whereas NK cells and ILC3 contributed to a lesser extent (16). Aryl Hydrocarbon Receptor (AHR) ligands that can either derived from diet or microbiota are important sensors of the intestinal immune cells and regulate ILC development and NK cell functions (52, 53).

Intestinal microbial manipulation has been recently proposed for the management and treatment of NAFLD. The administration of pro/prebiotics allows the reduction of intestinal permeability and inflammation (54). The Short-chain fatty acids (SCFAs), products of the fermentation of dietary fibers by the anaerobic intestinal microbiota, have beneficial effects on mammalian energy metabolism and foster anti-inflammatory immune responses. The SCFAs acetate, propionate, and butyrate are found in the intestinal tract and may cross the gut barrier to reach the liver through the portal vein blood (55). In addition, SCFAs could signal via FFAR3^+^ (GPR41) and FFAR2^+^ (GPR43) receptors in immune cells. Propionate and butyrate were described to attenuate pathogenic allergic Th2 responses by controlling DC hematopoiesis (56). Furthermore, SCFAs also enhanced protective CD8 T cell responses against flu infection and reduced neutrophil recruitment into the lung (57). Similarly, acetate has been shown to improve recall function of memory CD8^+^ T cells (58). Whether such metabolites regulate intestinal or liver NK cells and ILC1 functions is still an open question that could be addressed to propose new therapeutic approaches in NAFLD.

## Hepatic NK Cells and ILC1 in Nafld Development

The liver is mainly composed of hepatocytes (80% of liver cells) and non-parenchymal cells which include immune, endothelial, stellate and biliary cells. Liver NKp46^+^ cells account for approximately 10%-20% of total intrahepatic lymphocytes in mouse and almost 40–50% in human. In mice, liver NK cells are Eomes^+^ CD49b^+^ CD49a^−^ TRAIL^+/−^ while liver ILC1 are Eomes^−^ CD49b^−^ CD49a^+^ TRAIL^+^ (59, 60). Murine liver ILC1 have the potent ability to produce cytokines (IFN-γ, TNF-α, IL-2, GM-CSF) (20). It has been reported that mouse hepatic CD49a^+^ILC1 residing in the liver sinusoids contribute to the response against virus, haptens antigens and are endowed with memory properties (20, 61, 62).In human liver, the CD45^+^ Lin^−^ CD16^−^ NKG2A^−^ CD127^+^ CD161^+^ ILC population regroups CRTH2^+^ ILC2, ILC1, and NKp44^−^ ILC3 and NKp44^+^ ILC3 and is distinct from hepatic CD16^+^ or NKG2A^+^ NK cells (63). Moreover, equal proportion of liver CD56^bright^ and CD56^dim^ NK cells have been described among the total human liver NK cell population contrary to blood where the CD56^dim^ subset represents at least 90% of all peripheral blood NK cells. The liver CD56^bright^ population expresses Eomes but low levels of T-bet that should identify this population as conventional NK cells based on mouse studies. However, human hepatic CD56^bright^ expresses “tissue residency” markers such as CD69 and CXCR6 and thus are more similar to mouse ILC1/tr NK cells (64–66). Among the CD56^bright^ subset in the liver, a rare fraction (2.3%) are Eomes^−^ T-bet^+^ and expresses CD49a producing high levels of IFN-γ, TNF-α and GM-CSF (67). This small subset of liver CD56^bright^ CD49a^+^ cells represent a putative human counterpart to mouse hepatic CD49a^+^ ILC1/tr NK cells.

NK cells are involved in protective immune responses against hepatitis B and C virus infection or in limiting liver fibrosis and hepatocellular carcinoma (68). It is obvious that liver NK cells via TRAIL and/or NKG2D signaling have a major role in the removal of activated hepatic stellate cells in fibrotic liver or liver cancerous cells as reported in a tetrachloride mouse model of hepatic fibrosis (69, 70). We cannot rule out the contribution liver resident ILC1 in these earlier studies via either cytokine secretion or TRAIL-induced killing of target cells as recently described for activated NK cells (71). Hence, specific host protective roles are assigned to ILC during immune response. Hepatic ILC1 could contribute to the pathogenesis of chronic hepatitis B via pro-inflammatory cytokine production. ILC1 could then regulate cell responses against virus and intracellular bacteria (20, 72, 73). In that sense, Weizman et al. recently demonstrated that conventional liver resident XCR1^+^ dendritic cells produce IL-12 after MCMV infection which in turn promotes ILC1 production of IFN-γ (26). Inflammation is a major contributor of diet-induced liver complications. IL-15 is a pro-inflammatory cytokine essential for the homeostasis of NK cells but also NK-T cells, γδ T cells and memory CD8 T cells. IL-15 deficient mice are protected from obesity-associated metabolic disorders upon HFD challenge. IL-15 is involved in diet-induced lipid accumulation and hepatic inflammation (74). IL-15 decreases the hepatic expression of the fatty-acid oxidation transcription factor PPARα and promotes the lipid intake by regulating CD36 expression. IL-15 may also increase the pro-steatotic transcription factor PPARγ expression. IL-15 is a key cytokine for innate immune cells homeostasis during the development of hepatic steatosis. Therefore, we can speculate that the metabolic effect of IL-15 may be partly due to hepatic NK cell and/or ILC1 activities. Furthermore, a gene expression pattern of inflammatory markers such as IFN-γ, TNF-α, IL-18, CCL5, CXCL10 are upregulated in the NASH liver of morbidly obese patients compared to lean subjects that could be produced and/or could regulate type 1 ILC behavior (75, 76). IL-1α and IL-18 have been implicated in the transformation from murine steatosis to steatohepatitis (77). Adipokines (adiponectin, Ghrelin, and leptin) levels have been correlated to insulin resistance and their decrease could promote NAFLD evolution (78). It should be determined whether these adipokines could directly interact with NK/ILC1 subsets. Interestingly, hepatic osteopontin (OPN) gene expression is increased and is related to the severity of hepatic steatosis (79). OPN has been described as a chemotactic factor for dendritic cells, macrophages and regulates the lymphocyte phenotype (Th1 and Th17) (80, 81). It could be of interest to evaluate the contribution of OPN on liver type 1 ILC function as it is known that OPN promotes NK cell development and regulates NK cell homeostasis and functions (82, 83). However, it is needed to first identify which liver cells produce OPN during NAFLD development and whether liver type 1 ILC could respond to OPN to ascertain that OPN could be a new actor in the regulation of liver type 1 ILC functions.

**Figure 2:**
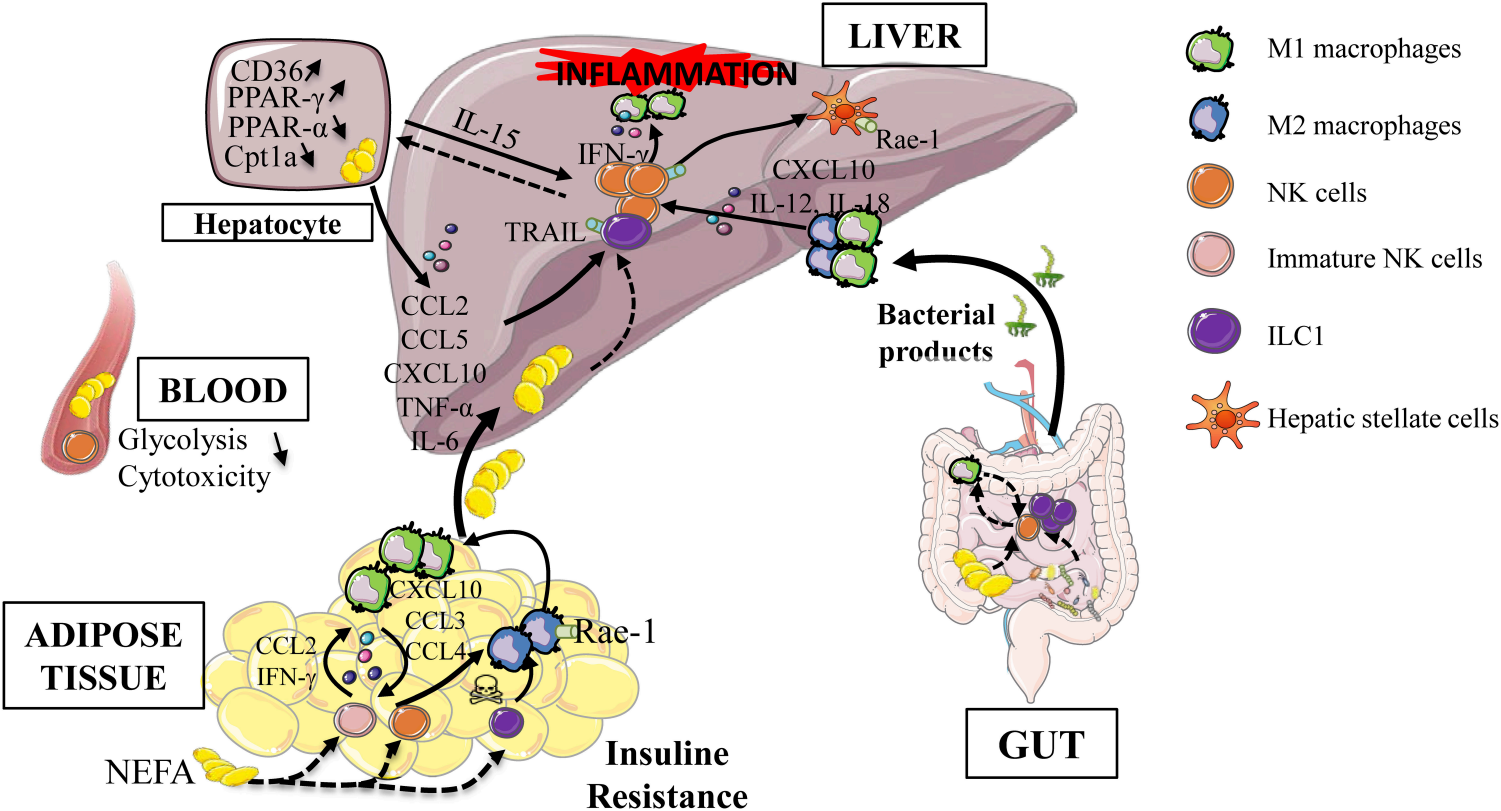
Type 1 ILC during obesity-induced liver inflammation. Obesity is associated with adipose tissue dysfunction, increased gut permeability and dysbiosis. These alterations are responsible for the release of bacterial products, the increase in endotoxin levels, the afflux of free fatty acids in the portal circulation as well the increase of *de novo* lipogenesis. These extra hepatic actors participate in the establishment of hepatic low grade inflammation. This leads to the activation of innate immune cells (group 1 ILC, dendritic cells, macrophages) and maintain the process of hepatic inflammation, via the production of inflammatory cytokines. All these events lead to a vicious cycle that aggravates liver injury, increases inflammation and thus promotes progression to more severe stages of the disease. Rae-1, retinoic acid early inductible−1; TRAIL, TNF-related apoptosis-inducing ligand; PPAR, peroxisome proliferator-activated receptor; Cpt1a, Carnitine Palmitoyltransferase 1a; NEFA, Non esterified fatty acid; FFA, Free fatty Acid; ILC, Innate Lymphoid Cells.

Myeloid and ILC1/NK cells are able to influence their effector functions by cell-cell contact and/or via cytokine production (26, 84, 85). In NAFLD, experimental evidences have shown that conversion of macrophage toward a pro-inflammatory phenotype contributes to disease severity (86, 87). Liver macrophages activation is mediated by translocated bacterial products due to intestinal permeability and also by the regulation of M1/M2 ratio. Indeed, In a murine model of steatohepatitis (methionine and choline deficient diet), IFN-γ-producing NK cells polarize macrophages toward M1 and NK cell depletion prevent fibrogenesis (88). In this mouse model, activated liver CD49b^+^ NK cells are increased whereas liver CD49a^+^ ILC1 are decreased suggesting a finely tuned interplay between these subsets. However, the elimination of NKp46^+^ cells required numerous injections of diphteria toxin, possibly generating local inflammation and macrophage cell death. Finally, type 1 ILC represent a major innate cell population in the liver and their ability to respond to various signals (stressed cells expressing NK receptor ligands, death receptors, IL-12/IL-18 cytokines, CCL5, CXCL10 chemokines…) and to interact with macrophages allow them to be a credible candidate involved in the development and severity of NAFLD (Figure 2).

## Metabolic Activities of NK Cells

To improve therapeutic strategies against cancer or microbial infections, efforts have recently been made to decipher metabolism regulation of immune cell functions. The basal activity of the metabolic checkpoint kinase mTOR (mammalian target of rapamycin) was shown to correlate with NK cell responsiveness and be enhanced upon IL-2 and IL-15 stimulation (89, 90). These studies pointed out the key role of mTOR signaling in the regulation of NK cell differentiation, granzyme B expression and IFN-γ production (91, 92). In addition, cytokine-stimulated NK cells upregulate the expression of Srebp 1/2 (Sterol-regulatory-element-binding proteins) target genes involved in cholesterol and fatty-acid metabolism (93). While lipid synthesis was not crucial for NK cell functions, Srepb unexpectedly regulated glucose metabolism which is critical for NK cell cytotoxicity and IFN-γ production. Therefore, mTOR and Srebp pathways may represent new targets to modulate NK cell functions. In addition, a recent report suggests a link between NK cell activity, mTOR, and OPN (83). OPN-KO NK cells harbored defective response to IL-15 with impaired upregulation of mTOR activity associated with defective NK cell development and functions. This phenotype is similar to mTOR deficient NK cells as shown by Marcais et al. (89). These results identify OPN as an intermediate in the IL-15 pathway that may regulate NK cell homeostasis and function through interactions with the mTOR pathway.

It is now well-established that obesity is associated with an increased risk of cancer and infections (94, 95). Recent evidences suggest that the impaired immunosurveillance observed in obese patients may be partly due to altered function of NK cells (91, 96). Peripheral blood NK cells from obese individuals displayed an activated phenotype and were hypo-responsive to stimulation *in vitro*. This exhausted phenotype highly correlated with body mass index and could explain the enhanced susceptibility of obese individuals to develop immune-related diseases such as cancers and infections (91). Recently, Michelet et al. demonstrated that the blunted anti-tumor response of NK cells during obesity is caused by the lipid accumulation in NK cells in a PPAR-dependent inhibition of the mTOR pathway (96). However, authors focused on spleen and blood NK cells without evaluating their tissue counterparts such as AT, intestine and liver, whose environmental changes are mostly impacted with obesity. How these changes affect the functions of tissue-specific ILC and regulate their cell-intrinsic metabolic pathways need to be addressed to better understand the mechanisms involved in NAFLD and therefore find new therapeutic targets.

## Conclusion

Here, we evaluated the contribution of NK cells and helper-like ILC1 in the setting of liver inflammation during NAFLD progression. Data highlight the contribution of adipose tissue and gut NK cells and ILC1 in the control of local inflammation and/or dysbiosis during obesity which in turn may contribute to the evolution of hepatic abnormalities. How metabolic changes associated with NASH progression impact ILC differentiation is still an open question. Further analyses are required to evaluate the effect of lipids and altered microbiota on tissue-resident ILC1 and NK cell function in targeted tissue during obesity. This will help to better appreciate the contribution of NK cells and ILC1 in the physiopathology of NAFLD.

## Author Contributions

CL, EV, TP, PG, and RG participated in the conception, interpretation, and writing the manuscript.

### Conflict of Interest Statement

The authors declare that the research was conducted in the absence of any commercial or financial relationships that could be construed as a potential conflict of interest.
